# Feedback Influences Discriminability and Attractiveness Components of Probability Weighting in Descriptive Choice Under Risk

**DOI:** 10.3389/fpsyg.2019.00962

**Published:** 2019-05-03

**Authors:** Shruti Goyal, Krishna P. Miyapuram

**Affiliations:** Centre for Cognitive and Brain Sciences, Indian Institute of Technology Gandhinagar, Gujarat, India

**Keywords:** risk, descriptive choice, feedback, probability weighting, prospect theory, discriminability, attractiveness

## Abstract

Our understanding of the decisions made under scenarios where both descriptive and experience-based information are available is very limited. Underweighting of small probabilities was observed in the gain domain when both description and experience were provided. The divergence observed from the prospect theory suggests a need for a separate or modified theory of decision making under risk. Recent studies suggest a possible role of probability weighting in the choice behavior under risk. We investigated both gain and loss domains with and without feedback for small and large probability conditions. We characterized the shape of the probability weighting function by a two-parameter functional form representing discriminability (concave-convex shape) and attractiveness (level of absolute weights relative to objective probability). We replicated a fourfold pattern of risk attitude on non-WEIRD population. We find that feedback leads to underweighting of small probabilities and overweighting of large probabilities in the gain domain and overall underweighting of probabilities in the loss domain. We find that underweighting of small probabilities is driven by changes in discriminability and attractiveness components in the gain domain and changes in the attractiveness component in the loss domain. We have interpreted the results by proposing an updated belief-based account of decisions under uncertainty in which feedback, when available, influences the probability weighting mediating the choice behavior.

## Introduction

Where to invest is a hard decision. Consider yourself making a choice between investing into the stock market, i.e., a risky prospect, or into a fixed deposit, i.e., a safe option. If you have past experience of investing into the stock market, you would probably make a different decision from someone new to investing. Where a novice would rely only on the descriptive information about current market trends, you would integrate market trends with your experience to arrive at a suitable decision. Many real life situations involve such risky decisions based on both descriptive and experience-based information. However, little is known about how decisions are made under such scenarios and how the two types of information are integrated (Barron et al., 2008). Relatively few studies have investigated risky decision making using description and experience paradigm (Newell and Rakow, 2007; Barron et al., 2008; Lejarraga and Gonzalez, 2011; Erev and Roth, 2014; Weiss-Cohen et al., 2016).

Decisions with risk are generally studied using descriptive paradigms or experience based paradigms. In descriptive paradigms, the experimenter informs the decision maker about an uncertain scenario with its possible outcomes and the corresponding outcome probabilities (Kahneman and Tversky, 1979; Tversky and Kahneman, 1992; Yechiam et al., 2005). In a typical experience based paradigm (Hertwig et al., 2004), the information about outcomes and outcome probabilities are learned either by making repeated choice on the options without any descriptive information followed by immediate feedback (Barron and Erev, 2003) or by sampling the available alternatives (Hertwig et al., 2004). Choices made from description are contrasted to choices made from experience (Barron and Erev, 2003; Hertwig et al., 2004; Erev et al., 2010; Rakow and Newell, 2010). Decisions from description reflect overweighting of small probabilities and underweighting of large probabilities (Kahneman and Tversky, 1979; Tversky and Kahneman, 1992; Trepel et al., 2005). Overweighting of probabilities refers to the decision weight of an event being larger than its objective probability. Similarly, underweighting of probabilities means that the decision weight of an event is higher than its objective probability (Barron and Yechiam, 2009). In contrast to decisions from description, decisions from experience reflect choices as if people underweight small and overweight large probabilities (Barron and Erev, 2003; Hertwig et al., 2004; Weber et al., 2004). This difference is referred to as “description-experience gap” (for review see Hertwig and Erev, 2009). The description-experience gap suggests a need for a separate or modified theory of decision making under risk based on experience (Hertwig et al., 2004; Weber et al., 2004).

A few studies have investigated scenarios where the decision maker has both descriptive and experience based information (Jessup et al., 2008; Lejarraga and Gonzalez, 2011; Weiss-Cohen et al., 2016). To study such scenarios, they have adopted the feedback paradigm from the experience only studies to the descriptive paradigm. Jessup et al. (2008) studied how experience in the form of feedback affects choices. They provided two groups of participants with a choice between a positive prospect (high probability: 0.8 or low probability: 0.05) and a sure option. In contrast to the no-feedback group, feedback was provided on every trial in the feedback group. They found an increase in the percentage of choosing the sure option for small probability prospects which suggests underweighting of small probabilities. Similar results can be observed in the study by Lejarraga and Gonzalez (2011) which explored the relative weight given to descriptive and experiential information for choices when both descriptive and experience-based information are available to the decision maker. They failed to observe risk attitude predicted by prospect theory for small and large probabilities. But results from their study suggest similar findings observed by Jessup et al. (2008), i.e., underweighting of small probabilities, indicated by the decrease in the percentage of risk-seeking choices. However, one of the limitations of these studies is that choices under loss domain have not been investigated for scenarios where both descriptive and experience-based information is available.

Probability estimation has been suggested to play an important role in mediating the effect of feedback on descriptive choices (Jessup et al., 2008; Wulff et al., 2018). Jessup et al. (2008) suggested that feedback drives subjective probability estimation toward objectivity. Wulff et al. (2018) performed a meta-analysis on the data from studies investigating experience based scenarios and found differences in probability weighting for experience-based choices and description based choices. However, the properties of the probability weighting function, namely, discriminability and attractiveness (Gonzales and Wu, 1999) have not been investigated. Gonzales and Wu (1999) proposed “two logically independent psychological properties that characterize the weighting function” (p. 139). Discriminability reflects the curvature of the probability weighting and attractiveness reflects its elevation. Discriminability is closely related to the concept of diminishing sensitivity (Tversky and Kahneman, 1992). It refers to the ability to differentiate between outcome probabilities. However, discriminability is not sufficient enough to explain degree of overweighting or underweighting of objective probabilities. Attractiveness informs us about the subjective appeal of probabilistic options overall. Attractiveness and discriminability are independent properties of probability weighting function. Discriminability can be explained using an example: consider a scenario where a researcher has to decide whether to spend more time on the manuscript that will increase the changes of acceptance by 5%. In which scenario would she invest more time on the manuscript, one where the existing chances of acceptance are believed to be 90% or one where it is believed to be 45%? (Gonzales and Wu, 1999). Improving the chances from 90 to 95% seems more substantial because our ability to differentiate between 90 and 95% is better compared to 30 and 35%. This example illustrates stronger sensitivity associated with change in probability around extreme events compared to that around moderate events. Attractiveness can be explained by another example such as buying life insurance for a bus travel (Baron, 2006). It means that the prospect itself is attractive reflecting overweighting of small probability. Investigating the properties of probability weighting function would inform if the underweighting reflected by the choices is due to changes in the degree of probability estimation (attractiveness) or due to differences in the weight associated with change in probability around extreme events (discriminability) or both. The current paper aims at investigating the parameters of probability weighting, specifically, discriminability and attractiveness, for the choices made under risk in gain and loss domains, when both descriptive and experience-based information are available to the decision maker.

Differences in experience based and description-based choices are explained by two theories that basically relate differences to problems at one of the two stages of the belief based account. The belief based account (Fox and Tversky, 1998) of decisions under uncertainty is a two-stage model that explains the process of decisions under uncertainty. The first stage corresponds to information acquisition, where decision makers aim to capture the probability distribution for each possible outcome. The second stage concerns the choice made given the probability distribution, governed by prospect theory. According to the information asymmetry account, information about the probability distribution in the two forms of decisions is not comparable (Rakow et al., 2008; Hadar and Fox, 2009). According to psychological account, the gap results because of the differences in the choice evaluation process (stage two) involved in the comparison of available alternatives to make decision making (Camilleri and Newell, 2009a, b). In the current study, the experience is provided on descriptive choices through feedback, making the probability information of the uncertain event available to the decision maker. This makes the first stage of belief based account comparable in both description only and description and experience together groups. According to the information asymmetry account, differences in choice are not expected. Any difference in the choice would result from differences in the evaluation component of belief based account. If there is a difference in the evaluation process involved between the two groups, the reason behind the difference needs to be addressed. The findings from previous literature suggest differences in probability weighting due to feedback (Jessup et al., 2008; Wulff et al., 2018). To address ‘how’ feedback influences descriptive choices, we have investigated the properties of probability weighting function.

The distortion in the probability weighting function because of feedback could be because of changes in attractiveness or discriminability, or both. The discriminability component would account for the over-weighting or under-weighting of small probabilities by increasing or decreasing our ability to discriminate between small probabilities relative to moderate events. Changes in the attractiveness component would account for over-weighting or under-weighting of small probabilities by making small probability prospects more or less attractive. Given that feedback influences choices under descriptive scenarios, it remains to be investigated which component(s) of probability weighting function mediate probability distortions.

Existing studies have used a repeated choice paradigm where participants have to make a choice on similar gambles repeatedly for a predefined number of trials to understand choices when both descriptive and experience-based information is available to the decision maker. A disadvantage of the repeated choice task is that participants have to make a choice on the same stimulus over and over again which is boring and repetitive. An adaptive procedure is a method where the stimulus presented on a given trial depends on the choice made by the participant on the previous trial (Jesteadt, 1980). The biggest advantage of the adaptive procedure is its task structure. In an adaptive procedure, a block starts with a few easy trials for the participants to understand the nature of the decision making task and the difficulty level increases automatically based on the participant performance (Jesteadt, 1980). For the stated advantages, the adaptive procedure was used to measure the certainty equivalent using the staircase method. The certainty equivalent is the sure amount of money that is equally attractive to a lottery. It corresponds to an indifference point where decision makers choose the sure option and the gamble equally often, i.e., they are indifferent between receiving the sure amount and playing a gamble.

The current paper has two major contributions. Firstly, we demonstrate differences in risk attitude for gain and loss conditions and for decisions where both descriptive and experience-based information are available. We found a risk-averse attitude for small probabilities and a risk-seeking attitude for large probabilities in the gain condition and a risk-seeking attitude for both small and large probabilities in the loss condition. Secondly, we propose that the behavioral differences between descriptive paradigm and description-experience together paradigm are mediated by differences in probability weighting. Our results indicate that underweighting of small probabilities is driven by changes in discriminability and attractiveness components in the gain domain and changes in the attractiveness component in the loss domain.

## Study 1: Replication

Given that Lejarraga and Gonzalez (2011) failed at replicating the results of prospect theory for description based scenario, we first investigated choices for description based scenario for gain and loss condition. We examined the fourfold pattern of risk attitude and assessed the robustness of overweighting small and underweighting large probabilities as proposed by prospect theory for decisions from description (Kahneman and Tversky, 1979). Given that previous studies have not implicated any differences in value function between descriptive and experience based scenarios, we restrict our analyses to the estimation of probability weighting function. This replication study formed the basis with respect to which the effect of feedback on descriptive choices would be evaluated.

### Methods

#### Participants

An *a priori* power analysis indicated a requirement of 24 subjects to have 96% power for detecting a large effect (Gpower: Erdfelder et al., 1996). Twenty Four university students participated in the study. The average age was 20.8 years (*SD* = 1.9). All the participants provided informed consent before starting the experiment.

#### Design and Procedure

The gambles used in the study are those used by Tversky and Kahneman (1992). The study used a within-subject design with two types of outcomes, gains [Indian Rupees (Rs.) 50 to 400] and losses [Indian Rupees (Rs.) 50 to 400] and nine pairs of outcome probabilities (50%,50%), (90%,10%), (10%,90%), (1%,99%), (99%,1%), (25%,75%), (75%,25%), (5%,95%), (95%,5%). The exchange rate at the time of experiment was 1 Indian Rupee = 0.015 US dollars. However, identical numerical values without applying the exchange rate were used, considering the amounts were reasonable. A total of 30 gambles were used in the experiment (Table 1). Outcome probabilities and outcome type was counterbalanced.

**TABLE 1:** Gain and Loss gambles (spots with ‘x’ refers to a gamble with the outcome value in the respective row and probability in the respective column) used in the experiment (adopted from Tversky and Kahneman, 1992) (Probability of the second outcome is given in the table. E.g., 99% chance of losing 400 in the bottom right gamble).

	Probability								
Outcomes	0.01	0.05	0.1	0.25	0.5	0.75	0.9	0.95	0.99
(0,50)			x		x		x		
(0,−50)			x		x		x		
(0,100)		x		x	x	x		x	
(0,−100)		x		x	x	x		x	
(0,200)	x		x		x		x		x
(0,−200)	x		x		x		x		x
(0,400)	x								x
(0,−400)	x								x

**FIGURE 1:**
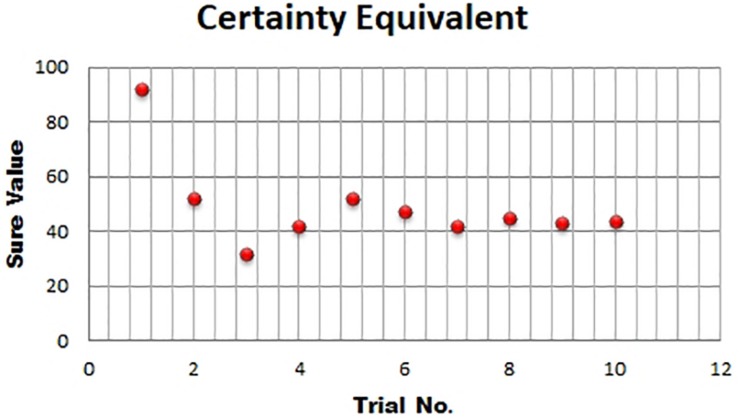
Certainty Equivalent of a gamble that yields a gain of Rs. 90 with 50% probability or Rs. 10 with 50% probability. It starts with a choice between a gamble and a sure value. Selection of sure value leads to a decrease in the value of the sure option by half of the range of gamble. The procedure continues until the step size becomes less than or equal to 2.

To calculate certainty equivalents (CEs), we used the Parameter Estimation by Sequential Testing (PEST) method (Luce, 1999). It is a staircase procedure where the value of the sure option is increased or decreased depending on the choice made by the participant. The starting value of the sure option is kept close ( ± 2 Indian Rupees) to the lowest or highest outcome of the gamble. The first trial is to make the participant understand the nature of the decision-making task. The sure amount increases (decreases) by the step size if the gamble (safe option) is selected. The initial step size is set as half of the range of the gamble and it decreases by half as the choice switches from gamble to the sure option. The procedure continues until the step size becomes less than or equal to two (Figure 1). The same procedure was used by Christopoulos et al. (2009).

The experiment started with an instruction screen where information about the task was presented to the participant. They were told that they will be presented with a choice between a gamble and a sure option presented on left or right side of the screen in counterbalanced manner. They then select one of the options by using the ‘Z’ or the ‘M’ key for the option on the left and right of the screen respectively. They were also informed that there are two types of conditions in the experiment, gain and loss. Both the gamble and the sure option under these conditions are of the same type – either gain or loss. Breaks were provided after every block. Because we used an adaptive procedure, each block lasted for different number of trials for different participants based on their choices.

After the instruction screen, participants were presented with the two options (e.g., 90% chance to win Rs. 50 and 10% chance to win Rs. 0 against a 100% chance to win Rs. 2). Decisions were self-paced. On each trial, the amount of the sure option was manipulated depending on the choice made by the participant on the previous trial. Participants completed the task in an average of 60 min. No outcomes were shown at end of the trial. Thus, participants made decisions from description only. Median certainty equivalent scores for all the gambles are made available in the Supplementary  Material (Supplementary Table S1).

### Results

We calculated the percentage of participants exhibiting risk-seeking or risk-averse attitude in gain and loss domains for small and large probabilities. This analysis was performed to understand risk preferences of the participants. The percentage was calculated by comparing certainty equivalent scores of each participant for all the gambles with corresponding gambles expected value. Risk seeking choices were defined as choices where the certainty equivalent of a gamble was larger than the expected value of the gamble (Tversky and Kahneman, 1992). The percentage of participants seeking or avoiding risk was calculated separately for small probability ( < = 0.25) and large probability ( > = 0.5) gambles (Table 2). With large probability, the majority of the participants were risk-averse for gains and risk seeking for losses (Table 2). With small probability, the majority of the participants were risk seeking for gains and risk-averse for losses (Table 2).

**TABLE 2:** Percentage of Risk Seeking choices for outcome probabilities in gain and loss conditions.

Probability	Gain	Loss
Small (*p* < = 0.25)	70%	24%
Large (*p* > = 0.5)	10%	75%

The gambles used in the experiment were of the type (X, P; 0, 1-P). Different probability conditions had different gambles. To compare the results between probability conditions data was normalized. Raw CE scores were normalized by dividing them by the non-zero outcome (X) of the gamble (i.e., CE/X) (c.f. Tversky and Kahneman, 1992). We performed descriptive analyses and ANOVA on these normalized scores.

A repeated measures (2x2) analysis of variance was performed on normalized certainty equivalent scores (CE/X) to understand if there are differences in risk attitude for different outcome domains and outcome probabilities. Independent variables included the within-subject variables outcome domain, defined at two levels (gain and loss), and outcome probability, defined at two levels (small probability: *p* < = 0.25 and large probability: *p* > = 0.5). There was a significant main effect of outcome probability [*F*(1,23) = 127.87, *MSE* = 3.217, *p* = 0.000]. An interaction effect between outcome probability and outcome domain was found significant [*F*(1,23) = 12.795, *MSE* = 0.200, *p* = 0.002]. A paired sample *t*-test was performed to understand the data. Following comparisons were made using paired sample *t*-test: the difference between large probability gain and small probability gain, between large probability loss and small probability loss, between large probability gain and large probability loss and lastly, between small probability gain and small probability loss. On average, normalized certainty equivalent scores were larger for large probability gain condition (*M* = 0.46, *SD* = 0.25) than for small probability gain condition (*M* = 0.18, *SD* = 0.22), with significant difference [*t*(23) = 6.62, *p* = 0.000]. On average, normalized certainty equivalent scores were larger for large probability loss condition (*M* = 0.65, *SD* = 0.20) than for small probability loss condition (*M* = 0.19, *SE* = 0.15), with significant difference [*t*(23) = 11.164, *p* = 0.000]. On average, normalized certainty equivalent scores were smaller for large probability gain condition (*M* = 0.46, *SD* = 0.25) than for large probability loss condition (*M* = 0.65, *SD* = 0.20), with significant difference [*t*(23) = −2.86, *p* = 0.009]. There was no significant difference between small probability gain condition and small probability loss [*t*(23) = −0.14, *p* = 0.89].

To estimate the parameters of the probability weighting function, we used non-linear least square regression using the curve-fitting toolbox in Matlab (MathWorks, 2015). The median normalized certainty equivalent scores across the participants were fit against each probability condition (for details refer to Etchart-Vincent, 2004; Fox and Poldrack, 2009). Least square errors were minimized based on Levenberg–Marquardt algorithm (Marquardt, 1963). Parameters of the weighting function were estimated using two-parameter function (Lattimore et al., 1992), which has suggested to be better than single parameter function at accounting the choice variability (Trepel et al., 2005). Two-parameter function provides additional information about probability weighting. Gamma (γ) represents discriminability and delta (δ) represents attractiveness (Gonzales and Wu, 1999).

$$\delta \,p^{\gamma }\delta \,p^{\gamma }+{\left(1-p\right)}^{\gamma }$$

**FIGURE 2:**
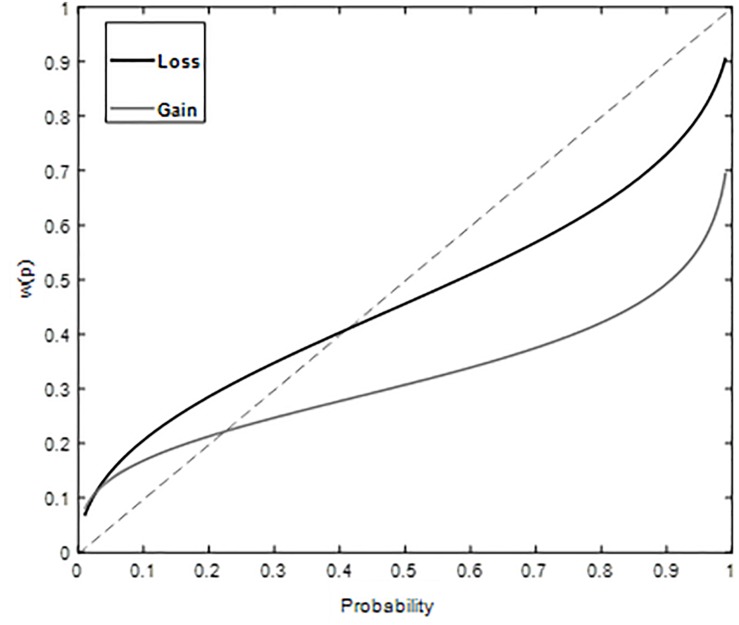
Weighted Probability for gains and losses plotted against probability in Study 1. Overweighting of small probabilities and underweighting of large probabilities is observed for both gain and loss domains.

The estimates of the probability weight using a two-parameter function were as follows – for gains: γ = 0.35, δ = 0.44 (Figure 2) and for losses: γ = 0.53, δ = 0.84 (Figure 2). These estimates correspond to an inverse S shape for both gains and losses, in-keeping with overweighting of small probabilities and underweighting of large probabilities. The goodness of fit gave high adjusted $R^{2}$ value, 0.94 (gain) and 0.97 (loss) for the two-parameter function.

## Study 2

This study investigated the effect of feedback on descriptive choices under risk using the same set of gambles as Study 1.

### Methods

#### Participants

An *a priori* power analysis indicated requirement of 24 subjects to have 96% power for detecting a large effect (Gpower: Erdfelder et al., 1996). Twenty four university students participated in the study. Their average age was 21.21 years (*SD* = 2.55). All the participants gave informed consent prior to the commencement of the experiment.

#### Design

A within-subject design was used. Gain and loss conditions along with outcome probabilities were manipulated as a within-subject factor. The experimental design was the same as that of Study 1 except that feedback was provided after every trial for 500 ms. The feedback informed participants about the outcome of the selected option. It was presented as “You won Rs. xx” or “You lost Rs. xx.” Median certainty equivalent scores for all the gambles are included in the Supplementary  Material (Supplementary Table S2).

### Results

We studied risk attitude as a function of probability using an ordinal analysis on the certainty equivalent scores of the participants. We calculated the percentage of participants exhibiting risk-seeking or risk-averse attitude in gain and loss domains for small and large probabilities. Percentage was calculated by comparing certainty equivalent scores of each participant for all the gambles with corresponding gambles expected value. Risk seeking choices were defined as choices where the certainty equivalent of a gamble was larger than the expected value of the gamble (Tversky and Kahneman, 1992). Table 3 shows that majority of the participants were risk averse for small probability gains except for 1% probability condition and majority of participants were risk seeking for large probability gains. Most of the participants were risk seeking in loss condition except for 1% probability condition.

**TABLE 3:** Percentage of Risk Seeking choices for small and large outcome probabilities in gain and loss conditions.

Probability	Gain	Loss
Small (*p* < = 0.25)	45%	74%
Large (*p* > = 0.5)	54%	77%

A repeated measures (2x2) analysis of variance was performed on normalized certainty equivalent scores to investigate differences in risk attitude for different outcome domains and outcome probabilities. Independent variables included the within-subject variable outcome domain (gain and loss) and outcome probability (small probability: *p* < = 0.25 and large probability: *p* > = 0.5). There was a significant main effect of outcome probability [*F*(1,23) = 799.50, *MSE* = 13.61, *p* = 0.000] but no effect of outcome domain (*p* = 0.46). Interaction effect was not found significant between outcome domain and outcome probability (*p* = 0.90). Paired *t*-test was performed between large and small probability conditions for gain and loss. On average, normalized certainty equivalent scores were smaller for small probability gain condition (*M* = 0.06, *SD* = 0.03) than for large probability gain condition (*M* = 0.82, *SD* = 0.22), with significant difference [*t*(23) = 17.038, *p* = 0.000]. On average, normalized certainty equivalent scores were smaller for small probability loss condition (*M* = 0.04, *SD* = 0.03) than for large probability loss condition (*M* = 0.79, *SD* = 0.18), with significant difference [*t*(23) = 21.0, *p* = 0.000]. Results suggest that the normalized certainty equivalents were significantly larger for large probability conditions compared to small probability conditions.

**FIGURE 3:**
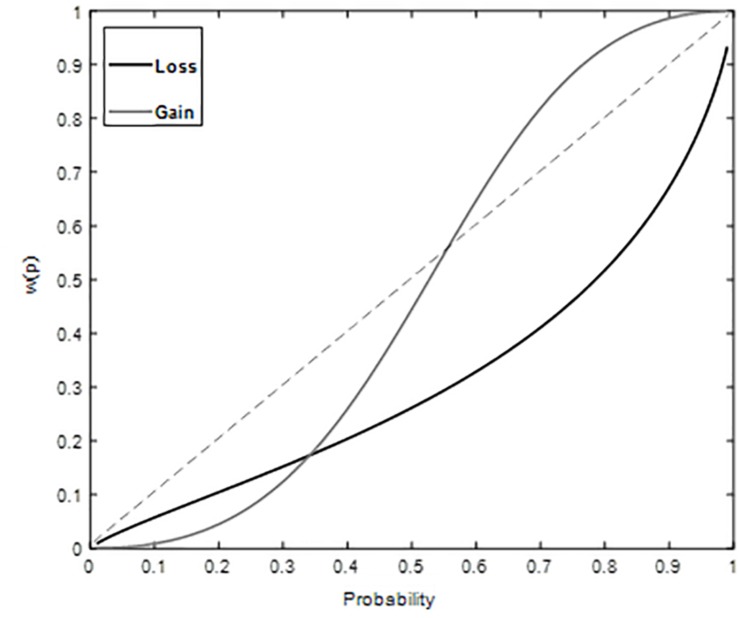
Weighted Probability for gains and losses plotted against probability Study 2. Underweighting of small probabilities is observed for both gain and loss conditions.

The estimates of the probability weight using two-parameter function were as follows, for gains: γ = 2.04, δ = 0.80 and for losses: γ = 0.80, δ = 0.35. We plotted weighted probability based on the obtained parameter estimates against probability (Figure 3), which no longer exhibits inverse S shape for both gains and losses that had been observed in decisions from description only (Replication Study above). Instead, with decisions from both description and experience participants showed underweighting of small probabilities in both gain and loss domains. Goodness of fit gave high adjusted *R*^2^, 0.97 (gain) and 0.94 (loss) for two parameter function.

## Comparing the Results of Study 1 and Study 2

A 2x2x2 mixed analysis of variance was performed. Independent variables included two within-subject variables, outcome domain (gain and loss) and outcome probability (small and large) and a between-subject variable, group (no-feedback/feedback). There was a main effect of group on normalized certainty equivalent sores [*F*(1,46) = 3.96, *MSE* = 0.14, *p* = 0.05]. There was an interaction effect between group, outcome probability and outcome domain [*F*(1,46) = 5.795, *MSE* = 0.109, *p* = 0.02], between group and probability [*F*(1,46) = 85.19, *MSE* = 1.80, *p* < 0.000] and between outcome probability and outcome domain [*F*(1,46) = 4.87, *MSE* = 0.09, *p* < 0.02]. To understand the data, paired sample *t*-test was performed between the following, large probability gain condition without feedback and large probability gain condition with feedback, small probability gain condition without feedback and small probability gain condition with feedback, large probability loss condition without feedback and large probability loss condition with feedback and small probability loss condition without feedback and small probability loss condition with feedback. There was a significant difference between large probability gain condition with feedback and without feedback [*t*(23) = 5.043, *p* = 0.000], large probability loss condition with and without feedback [*t*(23) = 3.46, *p* = 0.002], small probability gain condition with and without feedback [*t*(23) = −2.72, *p* = 0.01] and small probability loss condition with and without feedback [*t*(23) = −5.02, *p* = 0.000].

We compared the estimates of the probability weighting functions between the feedback group (decisions from description and experience) and no feedback group (decisions from description only) using confidence interval at 95% (Table 4). Results indicate a significant difference in attractiveness component between feedback and no-feedback groups for both gain and loss conditions. Overlapping confidence intervals indicate that the parameter estimate representing discriminability was not significantly different between feedback and no feedback groups for the loss condition but was significantly different for gain condition. This suggest that underweighting of small probabilities is derived by changes in both discriminability and attractiveness in the gain domain where as it is derived by changes in attractiveness in the loss domain.

**TABLE 4:** Parameter estimates of two-parameter probability weighting function for feedback and no-feedback groups.

Outcome type	Delta (δ) (Attractiveness) (95% confidence bounds)	Gamma (γ) (Discriminability) (95% confidence bounds)	Adj. R – square Two parameter
Gain			
(No feedback)	0.44 (0.37,0.52)	0.35 (0.29,0.42)	0.94
Loss			
(No feedback)	0.84 (0.70,0.98)	0.53 (0.44,0.62)	0.97
Gain			
(With feedback)	0.80 (0.54,1.07)	2.04 (1.04,3.04)	0.97
Loss			
(With feedback)	0.35 (0.22,0.49)	0.80 (0.58,1.02)	0.94

Diminishing sensitivity is manifested as possibility or certainty effect (Abdellaoui et al., 2005). To further elucidate our understanding of discriminability component of probability weighting function, *post hoc* analysis corresponding to possibility and certainty effects was performed. Possibility effect refers to the finding that more weight is associated with change in probability around a zero compared to same change around a moderate event (50% chances) (Abdellaoui, 2000; Etchart-Vincent, 2004). Certainty effect refers to the finding that more weight is associated with change in probability around a certain event (100% chances) compared to same change around a moderate event (50% chances) (Abdellaoui, 2000; Etchart-Vincent, 2004). It means that our ability to differentiate probabilities around extreme events is better compared to that around moderate events. Possibility effect was investigated by comparing the difference between weight given to 10% probability and weight given to 0% probability [i.e., w(0.1) – w(0)] to the difference between the weight given to 60% probability and weight given to 50% probability[i.e., w(0.6) –w(0.5)] (Etchart-Vincent, 2004). Certainty effect was investigated by comparing the difference between weight given to 100% probability and weight given to 90% probability [i.e., w(1) – w(0.9)] to the difference between the weight given to 60% probability and weight given to 50% probability [i.e., w(0.6) – w(0.5)] (Etchart-Vincent, 2004). In Study 1, possibility and certainty effects are observed in both gain and loss domains (Table 5). Results suggest that small probabilities are over-weighted and large probabilities are underweighted. In Study 2, possibility and certainty effects were not found in gain domains (Table 6). Certainty effect was not observed in loss domain and equal sensitivity to change around zero and moderate event is found in the loss domain (Table 6). Results suggest that the psychological impact of extreme probabilities has diminished when feedback was provided in case of decisions from description and experience together.

**TABLE 5:** Possibility and Certainty effect in gain and loss domains.

	Possibility effect	Certainty effect
	[w(0.1)–w(0) compared to w(0.6)–w(0.5)]	[w(1)–w(0.9) compared to w(0.6)–w(0.5)]
Gain	(0.17–0) > (0.34–0.31) 0.17 > 0.03	(1–0.49) > (0.34–0.31) 0.51 > 0.03
Loss	(0.21–0) > (0.51–0.46) 0.21 > 0.05	(1–0.73) > (0.51–0.46) 0.27 > 0.05
		

**TABLE 6:** Possibility and Certainty effect in gain and loss domain.

	Possibility effect	Certainty effect
	[w(0.1)–w(0) compared to w(0.6)–w(0.5)]	[w(1)–w(0.9) compared to w(0.6)–w(0.5)]
Gain	(0.01–0) < (0.65–0.44) 0.03 < 0.21	(1–0.99) < (0.65–0.44) 0.01 < 0.21
Loss	(0.07–0) < (0.37–0.30) 0.07 = 0.07	(1–0.71) > (0.37–0.30) 0.29 > 0.07

Reaction time data was explored for differences in information processing speed between feedback and no feedback groups. An independent sample *t*-test was performed to compare mean response times in no feedback and feedback groups. There was no significant difference in mean response time between the feedback (Mean RT = 3.11 ± 1.44 s) and no feedback group (Mean RT = 3.51 ± 1.40 s) [*t*(46) = 2.01, *p* = 0.26]. Results suggest that reaction times were not different between feedback group and no feedback group.

## Discussion

The purpose of the study was to investigate how feedback influences the probability weighting function in decisions from description. Through two experiments, we investigated positive and negative prospects for a range of probabilistic events. Results suggested that providing feedback on descriptive choices influences the attractiveness associated with gamble in both gain and loss domain making the gamble less attractive compared to that in the description only scenario. It reduces the sensitivity to change in probability (discriminability) around rare events in the gain domain.

The results for choices under descriptive paradigm replicated the predictions of prospect theory despite the population group being non-WEIRD (Western, Educated, Industrialized, Rich and Democratic) (Henrich et al., 2010). Results from Study 1 replicate overweighting of small probabilities and under-weighting of large probabilities in decisions from description. Risk seeking attitude was observed for small probability gain condition and risk-averse attitude for small probability loss condition. Results from Study 2 are in agreement with the choices observed by previous studies (Jessup et al., 2008; Lejarraga and Gonzalez, 2011; Weiss-Cohen et al., 2016) in gain domain. We find risk-averse attitude for small probabilities and risk-seeking attitude for large probabilities in gain condition and risk-seeking attitude for both small and large probabilities in loss condition. We observed underweighting of probabilities in the loss domains.

We investigated the predictions of information asymmetry account and psychological account to understand the processes involved in decisions under risk. According to information asymmetry account, no differences are expected between choices based on description only and choices based on both description and experience. However, our results suggest significant differences between the choices made. When feedback is provided, risk-averse attitude is observed in the gain domain and risk-seeking attitude in loss domain for small outcome probabilities. This suggests differences in the evaluation process involved in the two forms; decisions from description and decisions from both description and experience.

Parameters of the probability weighting functions were estimated using a two-parameter weighting function. The two parameters of the function have been associated with psychological traits, namely discriminability and attractiveness (Gonzales and Wu, 1999). Discriminability reflects the subject’s ability to differentiate between various outcome probabilities whereas attractiveness refers to the subjective appeal of probabilistic options overall. We found significant differences in both attractiveness and discriminability component between feedback and no feedback groups in the gain domain whereas significant differences in the discriminability component were only observed in the gain domain. Further investigation of possibility effect suggests that 10% change around zero has less weight associated with it compared to same change around moderate event in the gain domain. It means that the ability to differentiate between probabilities around zero becomes poor in the gain domain. In the loss domain, sensitivity to change in probability around zero becomes same as that around a moderate event. This means that the underweighting of small probabilities in the loss domain is no longer driven by sensitivity to rare event. Certainty effect analysis suggests that sensitivity around certain event has reversed in the gain domain but not in the loss domain. This suggests that firstly, underweighting of small probabilities observed when feedback is provided on descriptive choices is derived by poor discriminability around small probabilities and decreased attractiveness for small probability events in the gain domain. Secondly, the underweighting of small probabilities in loss domain is derived by decreased attractiveness for small probability events.

Results from the current study along with the existing literature (Jessup et al., 2008; Tobler et al., 2008; Wulff et al., 2018) suggest that feedback plays an important role in decision making. Repeated feedback can bring the probability weighting function close to linear (Tobler et al., 2008). Belief based account is the existing model that explains the process involved in decision making under risk. However, its predictive power is limited to the only description based choices and fails at explaining experience based choices. Our result suggests that experience as feedback influences probability weighting which mediates the choices made. When feedback is not available to the decision maker as in description based scenario, probabilities are weighted as per the predictions of prospect theory. We suggest adding a third stage, i.e., ‘feedback stage’ to the Belief based account to make it better at predicting choices for experience-based scenarios. The current study is limited to scenarios where both experience-based and descriptive information were available to the decision maker. Wulff et al. (2018) performed a meta-analysis on the data from studies that investigated experience based scenarios and found differences in probability weighting for experience-based choices and description based choices. Building on the results of Wulff et al. (2018), we argue that behavioral differences observed between descriptive paradigm and experience based paradigms are mediated by different probability weights associated with probabilities in the two paradigms.

The current study addresses the question of ‘how’ feedback influences decisions from description but leaves open the question of ‘why’ it does so. We suggest that emotional processes induced by feedback might provide an answer to the ‘why’ question. Future research may want to focus on the emotional processes involved in description and experience-based paradigm and its role in the description-experience gap.

## Ethics Statement

This study was carried out in accordance with the recommendations of IIT Gandhinagar Institutional Ethics Committee with written informed consent from all subjects.

## Author Contributions

SG was involved in theoretical formalization, study design, data collection, analysis, interpretation, and preparing the manuscript. KM was involved in study design, analysis, and preparing the manuscript.

## Conflict of Interest Statement

The authors declare that the research was conducted in the absence of any commercial or financial relationships that could be construed as a potential conflict of interest.[extraconflict]
