# Supplementary material for: Feedback Influences Discriminability and Attractiveness Components of Probability Weighting in Descriptive Choice Under Risk
Source: Front Psychol. 2019 May 3;10:962. doi: 10.3389/fpsyg.2019.00962 (PMC6509417; doi:10.3389/fpsyg.2019.00962)
Supplement: Supplementary file 1 [file Table_1.DOCX]

Supplementary Material

| Table S1: Median certainty equivalents for gains and losses (Study 1). | | | | | | | | | |
| --- | --- | --- | --- | --- | --- | --- | --- | --- | --- |
| **Probability (of the second outcome of each prospect)** | | | | | | | | | |
| **Outcome** | 0.01 | 0.05 | 0.1 | 0.25 | 0.5 | 0.75 | 0.9 | 0.95 | 0.99 |
| (0,50) |  |  | 11.5 |  | 18.5 |  | 23 |  |  |
| (0,-50) |  |  | -15.5 |  | -22 |  | -39 |  |  |
| (0,100) |  | 12.5 |  | 19 | 35.5 | 41.5 |  | 56.5 |  |
| (0,-100) |  | -13.5 |  | -24 | -45 | -64.5 |  | -79.5 |  |
| (0,200) | 26 |  | 23 |  | 56 |  | 80.5 |  | 138.5 |
| (0,-200) | -14.5 |  | -32 |  | -90 |  | -154.5 |  | -163 |
| (0,400) | 21.5 |  |  |  |  |  |  |  | 304 |
| (0,-400) | -24 |  |  |  |  |  |  |  | -347.5 |

| Table S2: Median certainty equivalents for gains and losses with feedback on descriptive choices (Study 2). | | | | | | | | | |
| --- | --- | --- | --- | --- | --- | --- | --- | --- | --- |
| **Probability (of the second outcome of each prospect)** | | | | | | | | | |
| **Outcome** | 0.01 | 0.05 | 0.1 | 0.25 | 0.5 | 0.75 | 0.9 | 0.95 | 0.99 |
| (0,50) |  |  | 7 |  | 19 |  | 41.5 |  |  |
| (0,-50) |  |  | -4 |  | -12.5 |  | -35.5 |  |  |
| (0,100) |  | 5 |  | 7 | 50 | 95.5 |  | 98 |  |
| (0,-100) |  | -4 |  | -4 | -25.5 | -60 |  | -87 |  |
| (0,200) | 7 |  | 10 |  | 86.5 |  | 195.5 |  | 197.5 |
| (0,-200) | -4 |  | -4 |  | -49.5 |  | -106.5 |  | -158 |
| (0,400) | 6 |  |  |  |  |  |  |  | 398 |
| (0,-400) | -4 |  |  |  |  |  |  |  | -375.5 |
